# Biosphere frontiers of subsurface life in the sedimented hydrothermal system of Guaymas Basin

**DOI:** 10.3389/fmicb.2014.00362

**Published:** 2014-07-31

**Authors:** Andreas Teske, Amy V. Callaghan, Douglas E. LaRowe

**Affiliations:** ^1^Department of Marine Sciences, University of North Carolina at Chapel HillChapel Hill, NC, USA; ^2^Department of Microbiology and Plant Biology, University of OklahomaNorman, OK, USA; ^3^Department of Earth Sciences, University of Southern CaliforniaLos Angeles, CA, USA

**Keywords:** subsurface, Guaymas Basin, hydrothermal, methane, alkane oxidation, energy metabolism

## Abstract

Temperature is one of the key constraints on the spatial extent, physiological and phylogenetic diversity, and biogeochemical function of subsurface life. A model system to explore these interrelationships should offer a suitable range of geochemical regimes, carbon substrates and temperature gradients under which microbial life can generate energy and sustain itself. In this theory and hypothesis article, we make the case for the hydrothermally heated sediments of Guaymas Basin in the Gulf of California as a suitable model system where extensive temperature and geochemical gradients create distinct niches for active microbial populations in the hydrothermally influenced sedimentary subsurface that in turn intercept and process hydrothermally generated carbon sources. We synthesize the evidence for high-temperature microbial methane cycling and sulfate reduction at Guaymas Basin – with an eye on sulfate-dependent oxidation of abundant alkanes – and demonstrate the energetic feasibility of these latter types of deep subsurface life in previously drilled Guaymas Basin locations of Deep-Sea Drilling Project 64.

## THE GUAYMAS MODEL SYSTEM

The Guaymas Basin in the Gulf of California is a young marginal rift basin characterized by active seafloor spreading and rapid deposition of organic-rich sediments from highly productive overlying waters. The juxtaposition of active seafloor spreading and thick sedimentary sequences has created a dynamic environment where tightly linked physical, chemical, and biological processes regulate the cycling of sedimentary carbon. In Guaymas Basin, deeply emplaced volcanic sills originating at the spreading center have indurated and hydrothermally altered their surrounding sediment matrix, and continue to shape hydrothermal circulation patterns today (Einsele et al., 1980; Lonsdale and Becker, 1985). Buried organic matter is transformed quickly into hydrocarbons; Guaymas Basin hydrocarbons are young enough to be ^14^C-dated and have an average radiocarbon age of approximately 5000 years (Peter et al., 1991). Hydrothermal pyrolysis transforms and mobilizes a major proportion of subsurface carbon sources: the organic carbon content of approx. 3–4 wt% in surficial Guaymas Basin sediments (De la Lanza-Espino and Soto, 1999) is reduced to 1–2% in subsurface sediments below sills (Rullkötter et al., 1982; Simoneit and Bode, 1982). Hydrothermal alteration of buried sedimentary organic matter generates petroleum compounds including complex mixtures of linear, branched and cycloalkanes, hopanes, steranes, diasteranes, olefins, and polynuclear aromatic hydrocarbons (PAHs; Simoneit and Lonsdale, 1982; Kawka and Simoneit, 1987; Didyk and Simoneit, 1989), low-molecular weight alkanes (Bazylinski et al., 1988), organic acids (Martens, 1990), and ammonia (Von Damm et al., 1985). This mixture of substrates permeates the sediments and sustains extensive microbial communities that – among other processes – mediate methanogenesis (Welhan, 1988), methane oxidation (Kallmeyer and Boetius, 2004), and sulfate reduction (Jørgensen et al., 1990; Jørgensen et al., 1992; Elsgaard et al., 1994; Weber and Jørgensen, 2002; Kallmeyer et al., 2003). Hydrothermal mobilization also re-injects buried carbon, esp. methane, into the biosphere, a process with climate history relevance (Lizarralde et al., 2011).

**FIGURE 1:**
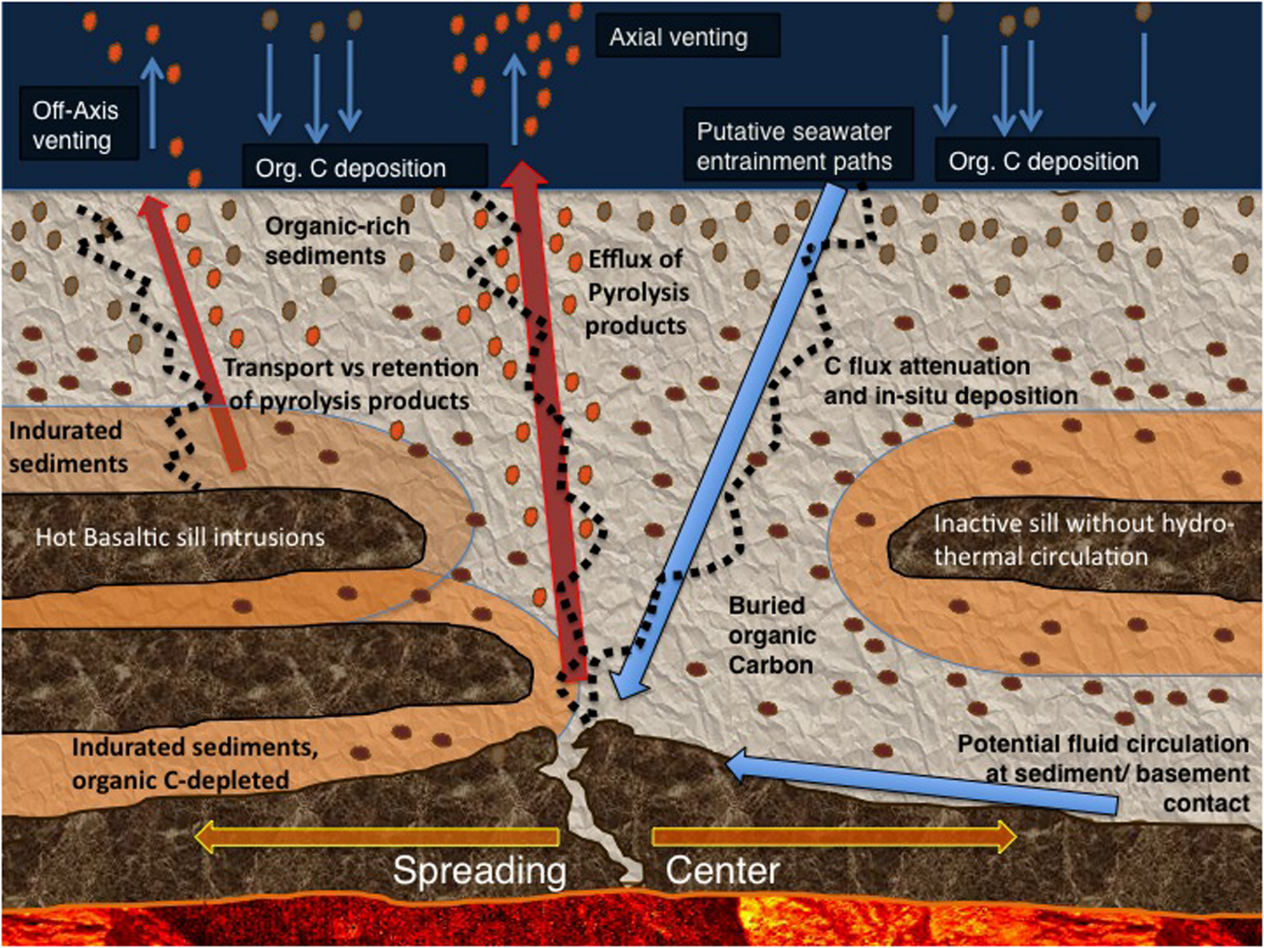
**Schematic representation of the Guaymas Basin subsurface with deep basement, sills, sediments, marine sedimentation and organic carbon input (brown drops), immobilized carbon in the sediment (dark-brown horizontal pellets), volatile pyrolysis products (orange drops), and hypothetical fluid flow pathways (arrows).** The black dotted lines indicate potential flow paths along fracture lines in the sediments; actually existing flow paths vary in location and vertical extent.

Guaymas Basin provides a classic example for young spreading centers that are often thickly sedimented due to their proximity to terrigenous sediment sources and the influence of coastal upwelling; modern examples include the Red Sea, the Gulf of Aden, the South China Sea, the East Sea/Sea of Japan, and the Aegean Sea. The interplay of geochemical, thermal and microbial forces that mobilize and assimilate carbon in the Guaymas Basin sediments provides a model system for exploring the extent, activity, biogeography and metabolic capabilities of subsurface microbial life within the extensive chemical and physical gradients of a young spreading center (**Figure 1**). Also, hydrocarbon-rich Guaymas Basin has particular promise for in-depth investigations of anaerobic hydrocarbon transformation, and the diversity and evolution of hydrocarbon-degrading microorganisms and pathways. Microbes capable of intercepting, oxidizing or assimilating hydrocarbons could attenuate the hydrothermally catalyzed mobilization and loss of buried carbon from hydrothermal sediments (Lizarralde et al., 2011). Yet, the extent and function of subsurface life in Guaymas Basin has not been probed since Leg 64 of the Deep-Sea Drilling Program (DSDP) targeted the massive sediments of Guaymas Basin (Curray et al., 1979; Curray and Moore, 1982) and demonstrated microbial methanogenesis in the deep sediment column (Galimov and Simoneit, 1982; Oremland et al., 1982).

## THE SURFACE LAYER

Diverse and abundant microbial life in the surficial hydrothermal sediments of Guaymas Basin is well-documented (reviewed in Amend and Teske, 2005). This survey of temperature and chemical regimes of Guaymas Basin starts at the sediment surface, where conspicuous microbial mats provide the highly visible surface expression of complex subsurface processes. Here, cold deep-sea bottom water supplies oxygen and nitrate; these oxidants permeate the surficial sediments not only by molecular diffusion, but are entrained by hydrothermal circulation (Gundersen et al., 1992). The nitrate influx into the upper sediment layers sustains active denitrifying microbial communities, which are inhibited by sulfide exposure (Bowles et al., 2012). Microbial mats of filamentous sulfur-oxidizing bacteria (family *Beggiatoaceae*) cover the seafloor in conspicuous patches (Jannasch et al., 1989). In shipboard experiments with fresh microbial mat samples, these bacteria take up autotrophic dissolved inorganic carbon (DIC), preferentially at moderate temperatures around 20°C (Nelson et al., 1989). These mats grow specifically in the areas where sulfidic fluids rich in DIC, methane, and low-molecular-weight organic compounds form gradients within the porewater right underneath the sediment surface, indicating near-surface consumption of these substrates (McKay et al., 2012). *In situ* temperature measurements in and beneath these oxygen- and nitrate-dependent sulfur-oxidizing mats indicate cool temperatures (near 10–15°C) right at the sediment surface within the mat, although the temperatures within the underlying sediments rise quickly and can reach >100°C within 30 cm depth (McKay et al., 2012). Genome sequencing of individual filaments supports a sulfur-oxidizing, nitrate-respiring metabolism with both autotrophic and heterotrophic capabilities (MacGregor et al., 2013a,b,c).

## THE MICROBIAL GAUNTLET

**FIGURE 2:**
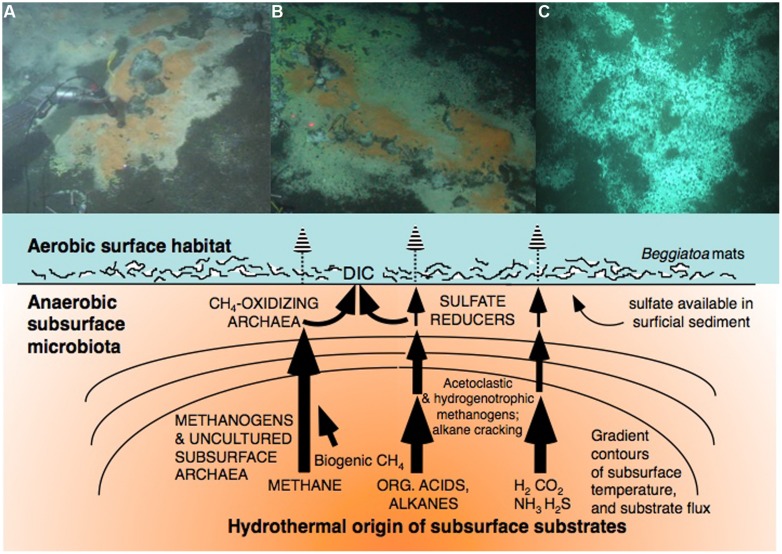
**The microbial gauntlet modifies the fluxes of deep carbon and gasses toward the sediment surface.** The top row shows examples for sulfur-oxidizing microbial mats dominated by filamentous *Beggiatoaceae* at well-documented hydrothermal seepage hot spots on-axis (**A**, Biddle et al., 2012; **B**, McKay et al., 2012) and off-axis in Guaymas Basin (**C**, Lizarralde et al., 2011). The *Beggiatoaceae* mats intercept DIC for autotrophic assimilation, and sulfide for oxidation with nitrate as the electron acceptor.

To map the unexplored taxonomic and physiological diversity of subsurface life in Guaymas Basin in relation to key chemical and thermal controls, the concept of the “Microbial Gauntlet” is helpful: Hydrothermally transformed organic substrates, or pyrolysis products from the hot end of hydrothermal gradients, are gradually processed and assimilated by subsurface microbial communities that populate the subsurface environment as soon as *in situ* temperatures and electron acceptor availability become compatible with their physiological requirements; microbial processing continues with cooler temperatures and increased electron acceptor availability towards the sediment surface, where seepage of hydrothermal fluids fuels abundant microbial mats (**Figure 2**). In this view, the microbial mats of Guaymas Basin represent the last stage of the microbial gauntlet in the sediments, as they intercept energy and carbon sources from hydrothermally active sediment and modulate microbial oxidation and carbon assimilation processes at the sediment/water interface.

## HIGH-TEMPERATURE METHANE AND SULFUR CYCLING

The microbial processes that generate, consume or modify the carbon and energy sources that are available at the sediment surface (for example sulfide, DIC and methane) extend into the hydrothermal sediments of Guaymas Basin. Given the strong hydrothermal gradients at Guaymas Basin, it is likely that the depth range of microbial processes is to a large part determined by temperature. By extrapolating from current evidence for microorganisms and microbial processes in surficial sediments (summarized in the following paragraphs), we hypothesize that the subsurface is permeated most deeply by microbial groups that tolerate maximal *in situ* temperatures and extremely reduced redox regimes. This is the domain of microbial methane and sulfur cycling; the temperature limits on these processes are outlined here.

Methanogens in Guaymas Basin sediments include numerous hyperthermophilic lineages, with the well-studied examples *Methanopyrus kandleri*, which has a growth limit at 110°C (Kurr et al., 1991; near 120°C under deep-sea *in situ* pressure, Takai et al., 2008), and *Methanocaldococcus jannaschii* and related strains growing at temperatures of 80–90°C (Jones et al., 1983, 1989; Jeanthon et al., 1999). Microbial community analyses, *in situ* temperature gradients, and porewater geochemistry from pushcores of surficial sediments have detected specific, phylogenetically distinct ANME-1 populations of methanotrophic archaea in high-temperature sediments at hyperthermophilic *in situ* temperatures (ranging from ca. 60 to >90°C), and concomitant δ^13^C-CH_4_ and DIC signatures of biogenic methane oxidation (Biddle et al., 2012). These high-temperature ANMEs are not limited to Guaymas Basin, but are widespread in methane-rich hydrothermal fluids at different vent sites (Merkel et al., 2012). Laboratory incubations and enrichments of sulfate-dependent, anaerobic methane oxidizing microbial populations from Guaymas Basin indicated a temperature limit of ca. 70°C (although with different ANME-1 populations), and preferred temperatures around 50–60°C for thermophilic methane oxidizers (Holler et al., 2011). High-pressure incubations with Guaymas sediment even showed low methane-oxidizing activities near 80°C (Kallmeyer and Boetius, 2004). When comparing the temperature ranges for methanogenesis and anaerobic (sulfate-dependent) methane oxidation, methanogenesis persists into higher temperatures (80–110°C) than methane oxidation; the latter reactions might also be limited by the temperature ceiling of sulfate-reducing syntrophs that participate in methane oxidation (Holler et al., 2011).

The thermal habitat requirements for sulfate- and sulfur-reducing archaea show a similar, if less pronounced, temperature gap. Different strains and species of the archaeal sulfate reducer *Archaeoglobus* (also isolated from Guaymas Basin; Burggraf et al., 1990) have upper temperature limits near 85–90°C (reviewed in Amend and Teske, 2005). Consistent with this temperature range of cultured hyperthermophilic sulfate reducers, microbial sulfate reduction in Guaymas Basin sediments has been detected at temperatures near 90°C (Elsgaard et al., 1994; Jørgensen et al., 1990; but note a 120°C outlier, Jørgensen et al., 1992). In contrast, the elemental sulfur-reducing *Thermococcales* of Guaymas Basin include *Thermococcus* and *Pyrococcus* strains that grow at temperatures near 100°C (Jannasch et al., 1992; Huber et al., 1995; Canganella et al., 1998; Edgcomb et al., 2007; Teske et al., 2009; Wang et al., 2011), approx. 10–15°C higher than the sulfate reducers. Differences in the thermal stability of the cellular components and enzymes of sulfate vs. sulfur reduction might account for this gap. So far, these methane- and sulfur-cycling hyperthermophiles have been isolated from surficial hydrothermal sediments of Guaymas Basin; similar organisms may exist in the deep surface of Guaymas Basin, given the detection of *Thermococcales* in deep hot oil fields (L’Haridon et al., 1995), and, together with different ANME archaea, in very deep, geothermally heated marine sediments (Roussel et al., 2008).

## HIGH-TEMPERATURE HYDROCARBON OXIDATION

In the upper sediment column, towards the sediment surface, and under generally more moderate environmental conditions at off-axis locations, less extremophilic microbial groups will gain a foothold, increase overall microbial biomass and activity, and broaden the chemical spectrum of microbially catalyzed reactions. Here we call attention to the mesophilic and thermophilic sulfate-reducing bacteria that anaerobically oxidize the diverse hydrocarbons in Guaymas Basin sediments and play a key role in the selective degradation of specific substrate classes, such as alkanes (Bazylinski et al., 1988). Guaymas Basin has developed into a preeminent sampling area to enrich and isolate novel alkane- and aromatic-degrading sulfate reducers that use different biochemical strategies to harness energy from hydrocarbons. The first aromatics-oxidizing, sulfate-reducing isolate from Guaymas Basin sediments, *Desulfothermus naphthae* strain TD3, grows optimally between 55 and 65°C, and couples the oxidation of C_6_–C_16_ alkanes and 3-methyloctane to sulfate reduction, although its specific alkane activation and degradation mechanism remains unknown (Rüter et al., 1994; Ehrenreich, 1996). Shortly after the isolation of TD3, a benzene-utilizing enrichment culture was obtained from Guaymas sediments (Phelps et al., 1996), which contained phylotypes that affiliate with members of the *Desulfobacterium anilini* and *Desulfobacter* clusters (Phelps et al., 1998); one of these *Desulfobacterium* phylotypes (SB-21) is possibly involved in benzene activation (Oka et al., 2008). Although benzoate was detected as an intermediate in this enrichment culture, the source of the carboxyl group remains unidentified (Phelps et al., 2001). The sulfate-reducing strain EbS7, isolated from Guaymas sediments, is capable of ethylbenzene degradation via addition to fumarate (i.e., “fumarate addition”; Kniemeyer et al., 2003). More recently, several cultures utilizing gaseous alkanes at mesophilic and thermophilic conditions were established with Guaymas Basin sediments. The culture Propane60-GuB grows at 60°C on propane via fumarate addition, and phylogenetic analysis revealed that the dominant phylotype belongs to the genus *Desulfotomaculum* (Kniemeyer et al., 2007). From the same study, another enrichment culture that utilizes butane at 60°C yielded dominant phylotypes that appear to be closely related to a deeply branching bacterial lineage dominated by clones from Guaymas Basin and other deep-sea sediment sites (Teske et al., 2002; Dhillon et al., 2003; synonymous with “Hot seep cluster,” Holler et al., 2011). The phylogenetic affiliation of this cluster is a matter of debate; while it was included among the Deltaproteobacteria by Holler et al. (2011), repeated tests with multiphylum full-length 16S rRNA gene alignments indicated that this group forms its own deeply branching bacterial lineage (Teske et al., 2002; McKay, 2014).

Taken together, these studies indicate a mesophilic to thermophilic temperature range (up to 65°C) for alkane- and aromatics-degrading sulfate reducers in Guaymas Basin, and suggest that hydrocarbons can be microbially remineralized in moderately heated sediments. As a caveat, sulfate may not be strictly necessary when sulfate reducers that can utilize alkanes also grow in syntrophic consortia (Callaghan et al., 2012). Further, it has to be emphasized that the thermophilic spectrum of sulfate-reducing bacteria is still poorly known. For example, a comparative CARD–FISH hybridization study of Deltaproteobacteria in different cold seep sediments and in Guaymas Basin hydrothermal sediments indicated that Guaymas Basin harbored reduced proportions of *Desulfosarcinaceae* and *Desulfobulbaceae* but the highest proportion of unidentified Deltaproteobacteria that did not react with group-specific 16S rRNA hybridization probes (ca. 44%), suggesting unexplored deltaproteobacterial diversity in Guaymas Basin (Kleindienst et al., 2012). The recent description of a novel autotrophic and thermophilic sulfur-disproportionating *Deltaproteobacterium*, *Dissulfuribacter thermophilus*, underscores the unexhausted potential of hydrothermal sediments for pure culture research (Slobodkin et al., 2013).

Last but not least, we are also calling attention to the possibility that hydrocarbon oxidation can be carried out at substantially higher temperatures, in the hyperthermophilic spectrum near and above 80°C. Some hyperthermophilic archaea have been characterized with respect to the anaerobic activation of non-methane hydrocarbons. Although not isolated from Guaymas Basin, *Archaeoglobus fulgidus* strain VC-16 serves as a model archaeal strain of *n*-alkene (Khelifi et al., 2010) and alkane degradation (Khelifi et al., 2014). The hyperthermophilic archaeon *Ferroglobus placidus* strain AEDII12DO DSM 10642, isolated from a sand-water mixture collected near Porto di Levante, Vulcano, Italy (Hafenbradl et al., 1996), couples the reduction of Fe(III) to the oxidation of aromatic compounds, including benzene; it is proposed that the molecule is activated by carboxylation (Tor and Lovley, 2001; Holmes et al., 2011). *Thermococcus sibiricus*, isolated from a Siberian oil field, can grow anaerobically on hexadecane at 78°C, although the activation mechanism is still unknown (Mardanov et al., 2009). Given the isolation of *Archaeoglobus* spp. (Burggraf et al., 1990), and other hyperthermophilic archaea from Guaymas Basin, including *Thermococcus* spp, *Pyrococcus* (Jannasch et al., 1992; Huber et al., 1995; Canganella et al., 1998; Wang et al., 2011), and *Geoglobus* (Kashefi et al., 2002), Guaymas Basin serves as an obvious platform to reconcile the gaps in our understanding of anaerobic hydrocarbon transformation with the elucidation of the metabolic versatility of Archaea.

## BIOCHEMICAL POTENTIAL FOR HYDROCARBON OXIDATION

Guaymas Basin offers a test case where the temperature range not only of specific organisms but of their biochemistry can be tested; here we focus on sulfate-reducing alkane oxidation since the organisms, substrates and processes are well-documented in Guaymas Basin. Alkanes are among the least reactive hydrocarbons due to their strong C-H bonds. With respect to non-methane alkanes, biodegradation is often initiated via addition of alkanes to the double bond of fumarate (“fumarate addition”) to produce alkylsuccinates (Kropp et al., 2000). The process is analogous to the addition of aromatic hydrocarbons to fumarate by benzylsuccinate synthase (BSS; Leuthner et al., 1998). Fumarate addition has been reported for both linear alkanes in the C3–C16 range (for review see Callaghan, 2013a) and for cycloalkanes (Rios-Hernandez et al., 2003; Musat et al., 2010). This mechanism may also be involved in the activation of solid paraffins, although the requisite metabolites have not been detected (Callaghan et al., 2010; Davidova et al., 2011). The addition of alkanes to fumarate is presumably catalyzed by the glycyl radical enzyme alkylsuccinate synthase (ASS; Callaghan et al., 2008b), also known as methylalkylsuccinate synthase (MAS; Grundmann et al., 2008), which has been used as a metabolic biomarker of anaerobic alkane transformation in a variety of isolates, enrichment cultures, and hydrocarbon-impacted environments (Callaghan, 2013a). Alternative mechanisms of anaerobic alkane activation by sulfate-reducing bacteria include carboxylation of *n*-alkanes, previously proposed for the sulfate-reducing isolate, *Desulfococcus oleovorans* Hxd3, and a nitrate-reducing enrichment culture growing on *n*-hexadecane (So et al., 2003; Callaghan et al., 2009). Genome sequencing of *D. oleovorans* inferred an enzyme complex similar to ethylbenzene dehydrogenase (Callaghan et al., 2008a), and recent investigations of strain Hxd3 suggest that this enzyme may play a role in alkane activation via anaerobic hydroxylation (Sünwoldt et al., 2012; Heider and Schühle, 2013). If proven, this homolog of ethylbenzene dehydrogenase may serve as a new *in situ* biomarker of anaerobic alkane transformation. Guaymas Basin sediments and enrichments offer the opportunity to “bioprospect” for these diagnostic enzymes and their genes.

An important feature of the Guaymas Basin is the complex mixture of alkanes, including methane. Methane concentrations in Guaymas Basin vent fluids range from 12 to 16 mM (Welhan, 1988). The conspicuous isotopic signatures of anaerobic methane oxidation (AOM) in Guaymas Basin sediments coincide with gene-based detection of ANME archaea; microbial methane oxidation changes δ^13^C-CH_4_ from a background value of ca. -43‰in hydrothermal sediments towards -20or -15‰ (Biddle et al., 2012). Elucidating the mechanism(s) of AOM is considered to be the “holy grail” of anaerobic hydrocarbon metabolism because it would provide insight to the cycling of the most important hydrocarbon on Earth, particularly with respect to methane sinks. To date, three hypotheses regarding AOM include: (1) “reverse methanogenesis” (Zehnder and Brock, 1979; Hoehler et al., 1994; Hallam et al., 2004), which is catalyzed by a homolog of methyl-coenzyme M reductase (MCR; Krüger et al., 2003; Scheller et al., 2010), (2) “intra-aerobic denitrification,” in which dinitrogen and molecular oxygen are produced from two nitric oxide molecules, the oxygen being used to functionalize methane by a methane monooxygenase (Ettwig et al., 2010), and (3) AOM and sulfate reduction being catalyzed by anaerobic methanotrophic archaea (ANME-2) via the reduction of sulfate to zero-valent sulfur (S^0^) and possibly to sulfide (Milucka et al., 2012). Alternatively, the addition of non-methane alkanes to fumarate has also prompted speculation that methane could be functionalized via fumarate addition, and the thermodynamic constraints have been hotly debated (for review see Callaghan, 2013a). Interestingly, methylsuccinate has been detected in sulfate-reducing and methanogenic subsurface environments such as oilfields and coal beds (Duncan et al., 2009; Gieg et al., 2010; Wawrik et al., 2012). However, no studies have definitely linked the detection of methylsuccinate with genetic biomarkers or other evidence indicative of a fumarate addition pathway.

Due to the ubiquity of methane, the persistence of methanogenic activity even in deep sediments, and the high temperature ranges of microbial methanogenesis and methane oxidation, the Guaymas Basin sediments provide a model system to investigate these distinct methane oxidation pathways in the deep subsurface.

## DETECTING METABOLIC POTENTIAL AND ACTIVITY IN GUAYMAS BASIN

Metabolite profiling, or metabolomics, has been a powerful tool used to elucidate the requisite metabolic pathways of anaerobic hydrocarbon activation (Callaghan, 2013b). Metabolomic studies and the discovery of enzymes involved in anaerobic hydrocarbon activation were together highly relevant for investigations of both natural and engineered systems, namely *in situ* investigations of contaminated groundwater and oil production water (Callaghan, 2013b). With a growing database of metabolic biomarkers and functional key genes in hand, more challenging environments are now being explored, such as coal-beds (Wawrik et al., 2012), the deep-sea sediments of the Gulf of Mexico (Kimes et al., 2013), and the oil-soaked sands associated with the Deepwater Horizon oil spill (Aeppli et al., 2012). Thus far, only a few studies have investigated Guaymas Basin for marker genes of anaerobic hydrocarbon degradation. In sediment samples from below a microbial mat in the Guaymas Basin (collected on Alvin dive 4573), two ASS genotypes (*assA*) and two (2-naphthylmethyl)succinate synthase genotypes (*nmsA*) were detected using targeted primers (von Netzer et al., 2013). In another study, metagenome analysis of an oil-immersed chimney in Guaymas Basin resulted in the detection of genes involved in anaerobic hydrocarbon activation (*assA*, *bssA*, and ethylbenzene dehydrogenase), although some inconsistencies in the descriptions of the model organisms and their hydrocarbon activation genes have to be noted (He et al., 2013). These functional gene surveys have focused mainly on enzymes catalyzing fumarate addition or anaerobic hydroxylation. However, with the recent discoveries of other putative marker genes such as naphthalene carboxylase (Bergmann et al., 2011; Mouttaki et al., 2012) and benzene carboxylase (Abu Laban et al., 2010; Holmes et al., 2011), the potential for different hydrocarbon activation strategies can be investigated *in situ* via metagenomic and/or metatranscriptomic approaches. Although Guaymas Basin has not been mined *in situ* for anaerobic hydrocarbon intermediates via metabolite analysis, functional key genes provide targets for metabolite profiling efforts. Given recent advances in our understanding of the biochemical pathways that govern anaerobic hydrocarbon oxidation, we are well poised to exploit omic-based methodologies to address questions regarding the metabolic strategies of thermophiles and hyperthermophiles with respect to hydrocarbon oxidation.

## HIGH TEMPERATURE LIMITATIONS IN THE SUBSURFACE

We hypothesize that the temperature zonation of different microbial communities and biochemistries translates into a vertical gradient or downcore sequence. How deep are the sediment layers that combine hospitable geochemical regimes and temperatures? In hydrothermal hot spots at the rift axis, microbially compatible temperature regimes are often found only near the sediment surface; downcore temperatures can increase to above 100°C within push coring range, ca. 30–50 cm depth (Jørgensen et al., 1990, 1992; Weber and Jørgensen, 2002; Teske et al., 2009; McKay et al., 2012). Such highly compressed temperature gradients in hydrothermal sediments are problematic for biogeographical sampling designs, since the microbial communities in these small-scale sediment gradients show a high degree of connectivity (Meyer et al., 2013). To avoid these shortcomings and to ensure a better chance of capturing biogeographical zonation in the Guaymas sediment column, sampling campaigns should move off-axis into cooler sediments characterized by lower hydrothermal heat flow and more gradual temperature (and chemical) gradients. *In situ* temperature gradients of the Guaymas Basin subsurface have been measured during DSDP Leg 64 in borehole 477, located in the Southern Guaymas Rift (27°01.85 N, 111°23.93 W; 2003 m water depth) and hole 481, located at the southern end of the northern Guaymas Rift (27°15.18 N, 111°30.46 W; 1998 m water depth; Kelts et al., 1982). *In situ* borehole measurements for site 477 yielded temperatures of 50°C at 49 m (extrapolated equilibrium temperature) and 87°C at 168 mbsf near the bottom, after 16 h of equilibration. For site 481, *in situ* borehole measurements yielded 3.6°C at the sediment surface, 9.0°C at 42 m depth and 56.8°C at 330 m depth, extrapolated from two temperature logs at the bottom (26.2°C after 3.5 and 51.0°C after 20 h of equilibration; Shipboard Scientific Party, 1982). The *in situ* mineralogy in hole 481 (sill/sediment contact metamorphism) is generally associated with temperatures below 200°C, and the oxygen isotopic composition of recrystallized calcites near the sill contact at 170 m depth indicates temperatures of 130–170°C. At site 477, the observed greenschist facies metamorphism implies temperatures >200°C below ca. 150 m depth. The stability of silica phases suggested exposure to hydrothermal fluids near 300°C (Gieskes et al., 1982a,b; Kastner, 1982). Given the lower measured temperatures at hole 477, this hydrothermal regime must have cooled by the time of drilling. In context, the temperature measurements during DSDP leg 64 suggest that microbially compatible temperatures extend into much of the subsurface in Guaymas Basin, since drilling and sampling designs avoid the hot spreading center.

## ENERGETIC POTENTIAL OF GUAYMAS BASIN SEDIMENTS

One of the key factors limiting microbial activity in any environment is energy. In order to assess the energetic landscape in Guaymas Basin sediments, geochemical data describing a representative sample of these sediments, DSDP Leg 64 Site 481A, were used to compute the Gibbs energy of redox reactions known to support microbial communities. The potential catabolic reactions chosen (**Table 1**) were determined by combining the likely electron donors in this setting, methane and a suite of short-chain *n*-alkanes (Whelan et al., 1988), and the only electron acceptor reported, sulfate (Gieskes et al., 1982a).

**Table 1:** Reactions considered to provide energy for microorganisms in Guaymas basin sediments.

CH_4_ + SO_4_^2-^ + H^+^ HCO_3_^-^ + HS^-^ + H_2_O	
4C_2_H_6_ + 7SO_4_^2-^ 8HCO_3_^-^ + 7HS^-^ + 4H_2_O + H^+^	
2C_3_H_8_ + 5SO_4_^2-^ 6HCO_3_^-^ + 5HS^-^ + 2H_2_O +H^+^	
4C_4_H_10_ + 13SO_4_^2-^ 16HCO_3_^-^ + 13HS^-^ + 4H_2_O + 3H^+^
C_5_H_12_ + 4SO_4_^2-^ 5HCO_3_^-^ + 4HS^-^ + H_2_O + H^+^	
4C_6_H_14_ + 19SO_4_^2-^ 24HCO_3_^-^ + 19HS^-^ + 4H_2_O + 5H^+^
2C_7_H_16_ + 11SO_4_^2-^ 14HCO_3_^-^ + 11HS^-^ + 2H_2_O + 3H^+^
4C_8_H_18_ + 25SO_4_^2-^ 32HCO_3_^-^ + 25HS^-^ + 4H_2_O + 7H^+^

**FIGURE 3:**
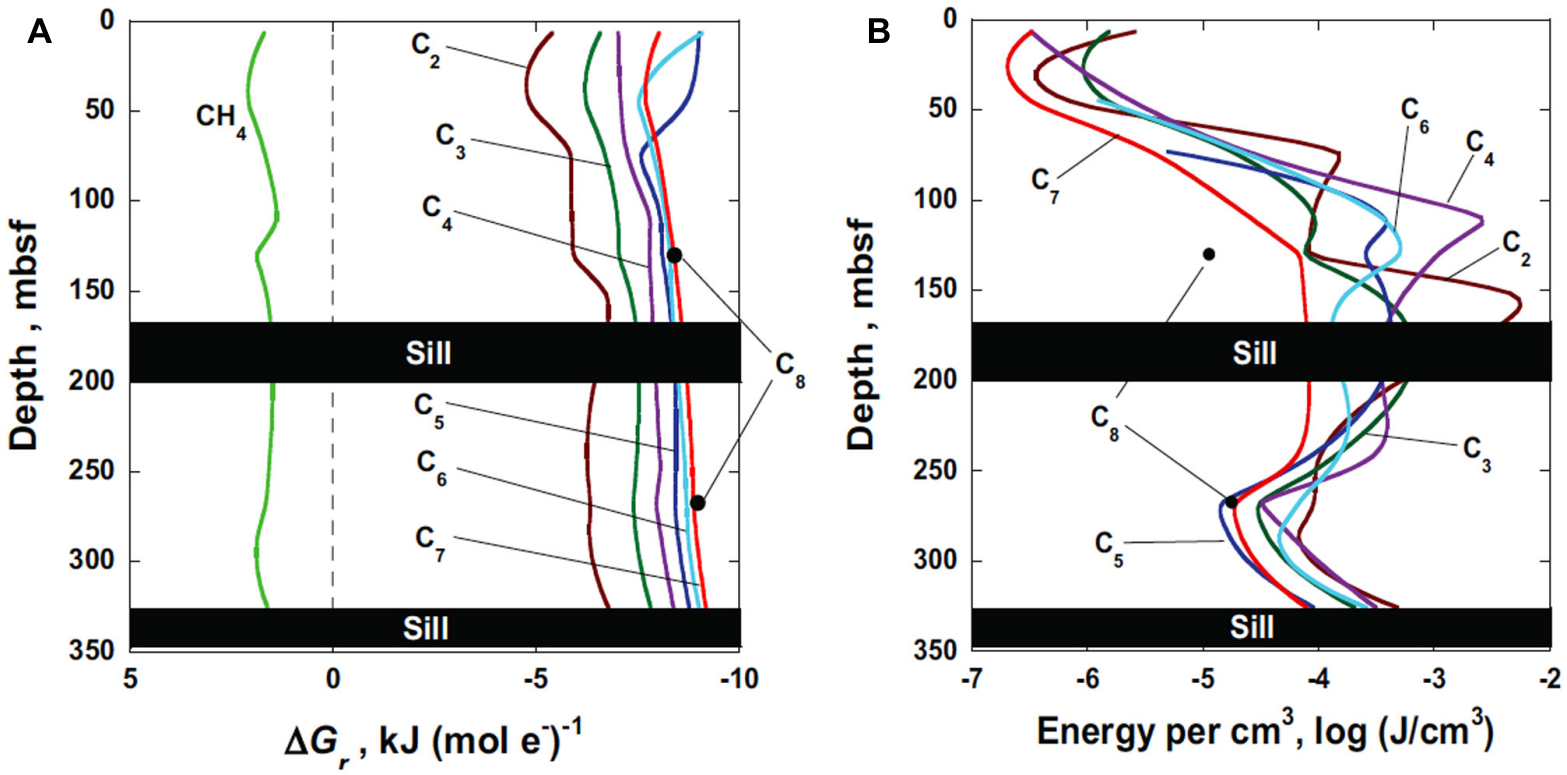
**(A)** Gibbs energy of sulfate reduction, Δ*G_r_*, coupled to methane and C_2_–C_8_ *n*-alkane oxidation in Guaymas Basin sediments (DSDP Leg 64, Hole 481A) in units of Joules per mole of electrons transferred, J (mol e^-^)^-1^. The reactions that these values of Δ*G_r_* refer to are listed in **Table 1**. **(B)** Energy availability in Guaymas Basin sediments (DSDP Leg 64, Hole 481A) in units of Joules per cubic centimeter of sediment, J cm^-3^, calculated using the Gibbs energy of reaction and the number of moles of the limiting substrate (*n*-alkane) in a cm^3^ of sediment.

The Gibbs energy of sulfate reduction coupled to the oxidation of methane and *n*-alkanes, C_2_–C_8_, in Guaymas Basin sediments are shown as a function of depth in **Figure 3A**. Values of Gibbs energies of reaction, Δ*G_r_*, are given in units of kilojoules per mole of electrons transferred, kJ (mol e^-^)^-1^ in order to facilitate comparison among the reactions. No results are shown at the depth intervals between 170 and 200 m and below 325 m due to the presence of impermeable sills. One of the more noticeable features in **Figure 3A** is that the anaerobic oxidation of methane by sulfate is thermodynamically not possible (positive values of Δ*G_r_*) throughout the sediment column (green line). However, the oxidation of all of the *n*-alkanes by sulfate have the potential to provide energy for microorganisms at all depths, ranging from about –5 to –9 kJ (mol e^-^)^-1^. At almost all depths, the longer-chain alkanes provide more energy per mole of electrons transferred. Furthermore, there is little variation in energy availability as a function of depth. Because the concentration of octane was only reported at two depths at Site 481A, values of Δ*G_r_* are only reported for these depths (black dots). For the other compounds, the lines represent calculations carried out at a number of depths along with interpolated values.

In **Figure 3B**, the Gibbs energies of the reactions listed in **Table 1** are plotted as energy densities – Joules per cm^3^ of sediment, units that are comparable to those typically used to report biomass in sediments, i.e., cells cm^-3^ (LaRowe and Amend, 2014). Although the same concentration data were used to generate values of Δ*G_r_* for Figures 3A,B the choice of normalizing the energy available per mole of electrons versus per cm^3^ yields considerably different patterns. First of all, it should be noted that the number of Joules available per mole of electrons transferred (**Figure 3A**) are several orders of magnitude larger than those available per cm^3^ of sediment. For instance, between about 10^-7^ and 10^-2^ J cm^-3^ are available for the reactions listed in **Table 1**. This is due to the fact that the concentration of alkanes at Site 481A are in the micro- to nanomolal range. That is, if all of the, e.g., heptane, were oxidized by sulfate in the shallowest portion of the sediment column (red line in **Figure 3B**), then a microbial community could only obtain about 10^-6.5^ J cm^-3^. This is substantially different than the ∼7500 J available per mole of electrons transferred for the same reaction (red line in **Figure 3A**).

Replotting the energetic yields of the microbial reactions (per mole of electrons transferred) as energy yield per sediment volume has interesting implications for subsurface life. For instance, whereas the energy available per mole of electrons transferred is relatively invariant with depth (**Figure 3A**), the amount of energy per cm^3^ (**Figure 3B**) changes by orders of magnitude as a function of depth. This can be seen for all of the reaction substrates: the energy available per cm^3^ from the sediment–seawater interface to the shallow sill increases by three to four orders of magnitude. Between the two sills, there is also about an order of magnitude difference in energy availability with depth. In addition, the order of which reaction provides the most energy changes from Figures 3A,B. The lighter alkanes tend to be more energy rich (**Figure 3B**), though there is considerable variation with depth due to crossing lines. Finally, one of the most compelling features of calculating Gibbs energies per cm^3^ that is not apparent in **Figure 3A**, is that the amount of energy available for microbial metabolism is largest near the sills. Whether or not microorganisms are there to exploit this energy is unknown, but the potential for larger biomass numbers near the sills is evident from the much larger amount of energy that is available there. This intriguing result suggests that the emplacement of sills in Guaymas Basin is associated with the generation of organic compounds that sulfate-reducing microorganisms are known to be able to metabolize for energy (Rüter et al., 1994; Kniemeyer et al., 2007).

## COMPUTATIONAL METHODS AND DATA SOURCES

The Gibbs energies of the reactions listed in **Table 1** were calculated using the revised HKF equation of state (Helgeson et al., 1981; Tanger and Helgeson, 1988; Shock et al., 1992), thermodynamic data from Shock and Helgeson (1988), Shock et al. (1989), Shock and Helgeson (1990), Sverjensky et al. (1997) and Schulte et al. (2001), the SUPCRT92 software package (Johnson et al., 1992) and compositional data from Curray and Moore (1982), Gieskes et al. (1982a) and Whelan et al. (1988). In order to carry out these calculations, the concentrations of sulfide (0.01 mmol) and DIC (15 mmol) were assumed based on porewater alkalinities for sites 477 and 481 (Gieskes et al., 1982a), and limited (qualitative) sulfide detection at site 481 (Shipboard Scientific Party, 1982). Activity coefficients were calculated using the extended Debye–Hückel equation (Helgeson, 1969) and the ionic strength of seawater (0.7 mol). Thermal gradient data generated previously (Curray and Moore, 1982) were used to calculate the temperature as a function of depth (from 2.9°C at 6.6 m depth to 44°C at 325 m). Porosity data generated from Einsele (1982) were used to convert the Gibbs energies of reaction per mole of substrate to Joules cm^-3^, using alkanes as the limiting substrate.

## OUTLOOK

The temperature tolerances and energy yields of microbial processes in the Guaymas Basin subsurface strongly suggest that microbial life is capable of colonizing these hydrothermally impacted sediments to considerable depths. While the hottest sediments in hydrothermal hot spots of the spreading center are likely to remain beyond the range of microbial life, moderately heated sediments at a judiciously chosen distance from channelized hydrothermal flow (such as those targeted on DSDP Leg 64) provide a very reasonable chance to explore the depth extent, density, genetic and functional diversity of subsurface life under hydrothermal controls.

## Conflict of Interest Statement

The authors declare that the research was conducted in the absence of any commercial or financial relationships that could be construed as a potential conflict of interest.
